# Laying the foundations of community engagement in Aboriginal health research: establishing a community reference group and terms of reference in a novel research field

**DOI:** 10.1186/s40900-022-00365-7

**Published:** 2022-08-04

**Authors:** Penny O’Brien, Ryan Prehn, Naz Rind, Ivan Lin, Peter F. M. Choong, Dawn Bessarab, Juli Coffin, Toni Mason, Michelle M. Dowsey, Samantha Bunzli

**Affiliations:** 1 Department of Surgery, St Vincent’s Hospital Melbourne, The University of Melbourne, Melbourne, VIC 3000 Australia; 2 Centre for Aboriginal Medical and Dental Health, The University of Western Australia, Perth, WA 6000 Australia; 3 Western Australian Centre for Rural Health, The University of Western Australia, Geraldton, WA 6530 Australia; 4 Ngangk Yira Institute for Change, Murdoch University, Murdoch, WA 6150 Australia; 5 ECCO Community Reference Group, Department of Surgery, St Vincent’s Hospital Melbourne, The University of Melbourne, Melbourne, VIC 3000 Australia

**Keywords:** Aboriginal health, Community engagement, Community reference group, Osteoarthritis, Research steering

## Abstract

**Background:**

Community engagement or community involvement in Aboriginal health research is a process that involves partnering, collaborating and involving Aboriginal and Torres Strait Islander people or potential research participants to empower them to have a say in how research with Aboriginal communities is conducted. In the context of Aboriginal health, this is particularly important so that researchers can respond to the priorities of the community under study and conduct research in a way that is respectful of Aboriginal cultural values and beliefs. One approach to incorporating the principals of community engagement and to ensure cultural oversight and guidance to projects is to engage a community reference group. The aim of this study was to describe the process of establishing an Aboriginal community reference group and terms of reference. The community reference group was established to guide the research activities of a newly formed research collaboration aiming to to develop osteoarthritis care that meets the needs of Aboriginal and Torres Strait Islander people in Australia.

**Methods:**

Adopting a Participatory Action Research approach, this two-phase study was conducted in Victoria, Australia. In phase one, semi-structured research yarns (a cultural form of conversation used as a data gathering tool) were conducted collaboratively by Aboriginal and non-Aboriginal co-investigators to explore Aboriginal health stakeholder perspectives on establishing a community reference group and terms of reference. In phase two, recommendations in phase one were identified to invite members to participate in the community reference group and to ratify the terms of reference through a focus group. Data were analyzed using a framework analysis approach.

**Results:**

Thirteen people (eight female, four male) participated in phase one. Participants represented diverse professional backgrounds including physiotherapy, nursing, general practice, health services management, hospital liaison, cultural safety education, health research and the arts. Three themes were identified in phase one; Recruitment and Representation (trust and relationships, in-house call-outs, broad-spectrum expertise and Aboriginal majority); Purpose (community engagement, research steering, knowledge dissemination and advocacy) and; Function and Logistics (frequency and format of meetings, size of group, roles and responsibilities, authority, communication and dissemination). In phase two, six Aboriginal people were invited to become members of the community reference group who recommended changes which were incorporated into the seven domains of the terms of reference.

**Conclusion:**

The findings of this study are captured in a 10-step framework which describes practical strategies for establishing a community reference group and terms of reference in Aboriginal health research.

**Supplementary Information:**

The online version contains supplementary material available at 10.1186/s40900-022-00365-7.

## Plain English Summary

Community engagement or community involvement in Aboriginal health research is a process that involves partnering with Aboriginal and Torres Strait Islander people or potential research participants to empower them to have a say in how research with Aboriginal communities is carried out. In the context of Aboriginal health, this is particularly important so that researchers can respond to the priorities of the community and conduct research in a way that respectful of Aboriginal cultural values and beliefs. One approach to community engagement in research is to form a community reference group to provide input to the research project. Although using a community reference group is considered to be an effective way to involve community members in research, often there are practical challenges in setting up and sustaining such a group. In this paper, we set out to describe an approach used to set up a community reference group for a new Aboriginal health research project exploring joint pain in Aboriginal and Torres Strait Islander people. This involved interviewing 13 health professionals and researchers (12 who identified as Aboriginal and one who identified as Aboriginal and Torres Strait Islander) about how to best go about setting up a community reference group. We used recommendations from these participants to inform who we approached to be members of the group and how the group would function. In describing the process we used to establish a community reference group, we were able to design a 10-step practical guide which may help other research groups who are looking to conduct new, ethical Aboriginal health research projects.

**Supplementary Information:**

The online version contains supplementary material available at 10.1186/s40900-022-00365-7.

## Background

Community engagement or community involvement in Aboriginal health research is a process that involves partnering, collaborating and involving Aboriginal and Torres Strait Islander people (respectfully Aboriginal people herewith, see Box 1) to empower them to have a say in how research with Aboriginal communities is conducted. It aims to protect and empower participating Aboriginal people and communities and is an ethical requirement in Aboriginal health research [1]. As with Indigenous peoples of other colonized countries such as Canada, the United States of America (USA) and New Zealand, Aboriginal people in Australia have endured significant health disparities as a result of the impact of colonization and associated race-based government policies [2, 3]. Although research provides an opportunity to address persistent health disparities, there remains concern that Indigenous peoples globally have been ‘researched to death’ without corresponding improvements in health [4]. One way to move forward is by embedding community engagement practices which involve Aboriginal people in all stages of health research from design to implementation and dissemination [5–7]. This has been described as an entry point to ‘decolonizing methodologies’ and in turn may ensure the ethical integrity and value of the research by shifting the focus away from researchers and to maximising community benefit [2, 6, 8].

**Box 1:** Terminology

Terminology regarding Aboriginal and Torres Strait Islander identity is varied and complex. In this paper, we use the term Aboriginal when referring to Aboriginal and Torres Strait Islander people, communities, co-researchers and participants in this study. No disrespect is intended to Torres Strait Islander people and we acknowledge the diversity of cultures of all Aboriginal, Torres Strait Islander and Aboriginal and Torres Strait Islander peoples in Australia. We use the term Indigenous when referring to global Indigenous populations or when referring to international concepts, such as research methods	

Based on the work of the Council for International Organizations of Medical Sciences, community engagement can be defined as ‘a process of engaging potential participants and communities in a meaningful participatory process that involves them in an early and sustained manner in the design, development, implementation and monitoring of research and in the dissemination of its results’ [5, 9]. Depending on the context, community engagement can include concepts such as community consultation, communication, education, participation, empowerment, collaboration and partnerships [10, 11]. Building partnerships with Aboriginal community representatives enables research which responds to community identified priorities, is guided by Aboriginal people and can facilitate decision making made in accordance with cultural values and beliefs. In addition to gaining insight to community identified priorities, researchers can gain a better understanding of the community under study which in turn informs and transforms the way in which we plan, develop and deliver health care. In a practical sense, engaging Aboriginal health stakeholders (i.e. anyone who has a ‘stake’ in the research, see Box 2) [12] such as Aboriginal health professionals, Aboriginal researchers and Aboriginal community members with lived experience of health conditions can together provide cultural brokerage and practical advice for non-Aboriginal researchers who can take on the role of facilitator [12, 13]. Involving stakeholders in research leads to research outcomes of greater quality and clinical relevance due to their being able to contribute unique perspectives and experiential expertise to the expertise of researchers [14–17]. This collaborative approach between non-Aboriginal researchers and Aboriginal researchers, services and communities is fundamental to informing, guiding and influencing how Aboriginal health care is designed and delivered and has the potential to help address persistent health disparities between Aboriginal and non-Aboriginal people [10, 18–20].

**Box 2:** Definitions

Aboriginal Community Controlled Health Organisation (ACCHO)	Aboriginal Community Controlled Health Organisations are incorporated Aboriginal organizations which provide primary health care services initiated and operated by Aboriginal community members. They provide comprehensive culturally secure health care to the community.
Acknowledgement of Country	An Acknowledgement of Country is an opportunity to acknowledge and show respect to Aboriginal and/or Torres Strait Islander people as Traditional Owners and ongoing custodians of Country. Acknowledgments are often made at the opening of events or the beginning of a meeting by acknowledging the Aboriginal nation and/or clan group name of which the event is taking place and acknowledging their cultures and long and continuing relationship with Country.
Community engagement	Also termed community involvement, community engagement in Aboriginal health research is a process that involves partnering, collaborating and involving Aboriginal community members or potential research participants to empower them to have a say in how research conducted with Aboriginal communities is conducted.
Cultural security	Cultural security can apply to both research processes and health care and occurs when research is conducted, or health services are offered, in a way that will not compromise the cultural rights, values, beliefs, knowledge systems and expectations of Aboriginal people [27].
Elder	An Aboriginal and/or Torres Strait Islander Elder is someone who is highly respected and recognized in their community as a custodian of cultural knowledge.
Stakeholders	Stakeholders were defined as anyone who has a ‘stake’ in the research, in particular those with important knowledge, experiences, expertise or views that should be taken into account [12]. In the context of the current study, stakeholders were Aboriginal community members who represented perspectives in Aboriginal health and Aboriginal health research or perspectives of Aboriginal people who experience osteoarthritis or living with someone who experiences osteoarthritis.
Sorry Business	Sorry Business is a term used to described Aboriginal cultural practices associated with death and grieving.

One approach used to incorporate the key elements of community engagement and involve Aboriginal people in Aboriginal health research is to engage a community reference group to provide oversight, input and cultural guidance to research projects [5, 20]. Community reference groups formalize the academic-community partnership by providing a mechanism which emphasizes information and power-sharing, mutual respect and reciprocity between community members and researchers [5, 21]. The roles and responsibilities of community reference groups vary from project to project, yet activities conducted by the group may include reviewing project documents and study materials, participating in community liaison and communicating community concerns, advocating for the rights of research participants, identifying community priorities and providing advice on study design, implementation and dissemination [5–7].

While the purpose of a community reference group in Aboriginal health research is to strengthen partnerships, previous research highlights both operational and conceptual challenges in maintaining ongoing community engagement through this strategy [5]. Challenges include unclear power dynamics or a lack of decision-making influence of the group, financial constraints, mistrust of non-Aboriginal researchers, time commitment, limited capacity of members and issues facilitating group discussions in a way that enables Aboriginal community members a strong voice [5, 6]. Failing to address such challenges can lead to weakened partnerships, skepticism about the role of the community reference group or concerns from members that their involvement is ‘tokenistic’ [6, 22]. As a guiding body intended to represent the views of the community, there are also inherent challenges surrounding the notion of representation, particularly when working in diverse populations. The question of whom and how communities should be represented is complex.

Despite being endorsed in policies articulated by national research organizations in Australia and internationally, there remains a lack of Aboriginal community engagement in research. A recent systematic review investigating patterns of community engagement in arthritis studies in Canada, USA, Australia and New Zealand found that the majority of arthritis research projects published do not involve Indigenous peoples at meaningful levels, leading to minimal benefit to the participants and communities involved [3]. Meaningful levels of engagement were defined in this review based on a spectrum of community engagement which includes: informing, consulting, involving, collaborating with; and empowering communities of interest [2, 23]. Furthermore, it has been observed by Indigenous health researchers in Canada that although there is interest from Aboriginal and non-Aboriginal researchers to enact the principles of community engagement whilst conducting Aboriginal health research, there is a general uncertainty on how to do so [2]. This is supported by increasing contributions to literature which describe ideas for improved ethical research and engagement and practices [2, 24, 25]. However, there is a lack of primary research which describes recommendations or practical guidance for health researchers and practitioners on how to engage Aboriginal people and communities in community engagement practices. Therefore, the aim of this study was to provide a practical example of how to establish a community reference group and terms of reference within a Participatory Action Research (PAR) framework [26]. By demonstrating this process within a novel area of Aboriginal health research, we also aimed to present practical recommendations within this context to inform future research groups.

## Project context and setting

### The ECCO collaboration, addressing a novel Aboriginal health area

The Enhancing Equity, Collaboration and Culturally secure Osteoarthritis care for Aboriginal Australians (ECCO) collaboration is a national inter-professional team of Aboriginal and non-Aboriginal health practitioners, health service staff, and research leaders that was established in response to an unaddressed health care gap; the mismatch between the burden of osteoarthritis and access to appropriate care. Cultural security in health care occurs when services are offered by the health system in a way that will not compromise the cultural rights, values, beliefs, knowledge systems and expectations of Aboriginal people [27]. The objective of the ECCO collaboration is to build an evidence-based model of osteoarthritis care that addresses the needs of Aboriginal people. Establishing a community reference group was a foundational step to building partnerships within the ECCO collaboration as well as between ECCO and external health services and Aboriginal community members.

## Research methods

### Ethical considerations

This project follows the National Health and Medical Research Council (NHMRC) guidelines for ethical conduct for research involving Aboriginal and Torres Strait Islander People and Communities [1]. Aboriginal community engagement and consultation was at the core of this project. Each of the six values of spirit and integrity, cultural continuity, equity, reciprocity, respect, and responsibility have been addressed in ethics applications approved by St Vincent’s Hospital Melbourne Human Research Ethics Committee [HREC185/19].

### Study design

This qualitative study was guided by the principle of cultural security (Box 2), which in the context of research refers to processes that ensure that research is conducted in a manner respectful of Aboriginal cultural values and beliefs [27]. This consideration is essential for ethical purposes [1], improves data quality and ensures that the interpretation incorporates an Aboriginal cultural lens [13, 27]. For example, culturally secure research methods such as yarning, an Aboriginal cultural form of conversation, were used as a data gathering tool [28]. Research yarning is acknowledged as being able to prioritize the lived experience and cultural context of Aboriginal participants [28]. Research yarning aligns with Aboriginal ways of knowing and doing, such as the use of storytelling. It ensures interviews and focus groups are informal, relaxed and requires the researcher to build a relationship that is accountable to Aboriginal people participating in the research [28].

The overarching theoretical framework was PAR [26]. Participatory Action Research is a framework for conducting research and generating knowledge which seeks to situate power within the research processes with those who are most affected by the research [29]. Participatory Action Research frameworks are considered particularly relevant for Indigenous peoples as the approach can help minimize the impact of ‘colonizing effects’ by shifting the power away from the dominant cultural perspective [26]. At the core of PAR is that power be deliberately shared between researchers and the researched by ensuring that those being researched are actively involved in the research process [26]. In this study, we were guided by the principals of PAR to establish a community reference group, who would then be engaged in an ongoing PAR research framework throughout all future Aboriginal health research activities conducted by the ECCO collaboration.

### Aboriginal capacity building

Central to this project and the ECCO collaboration more broadly, is building clinical and Aboriginal research capacity in musculoskeletal health. At the outset of this project, we appointed an Aboriginal co-investigator (NR) with a background in nursing and an interest in health and medical research. With no prior research experience, NR worked alongside a female qualitative researcher (PO) with extensive experience conducting interviews with diverse populations and a background in social science. PO supported NR to develop research skills, whilst NR was able to provide cultural guidance on the research activities and a connection to members of the local Aboriginal community. By the conclusion of this study, the ECCO collaboration had appointed a second, male Aboriginal co-investigator (RP) to ensure gender balance within the research team. With a background in sociology and Indigenous studies, RP also participated in training and professional development including qualitative data collection and analysis for the purpose of this project. RP also provided extensive cultural guidance and support with intercultural communication throughout the project. By working collaboratively with Aboriginal co-investigators, the research team ensured mutual benefit and reciprocity between Aboriginal and non-Aboriginal researchers.

### Participants and recruitment

To recruit members to our community reference group and to inform the development of the terms of reference, we started by interviewing participants from our target population. Our participants were key informants who represented different stakeholders. Stakeholders were defined as anyone who has a ‘stake’ in the research, in particular those with important knowledge, experiences, expertise or views that should be taken into account [12]. Eligible participants were Aboriginal and/or Torres Strait Islander adults residing in Victoria, Australia who spoke English and represented stakeholder perspectives in Aboriginal health and Aboriginal health research or perspectives of Aboriginal people who experience osteoarthritis. Initially, participants were purposively sampled by drawing on existing networks of project staff. This was augmented by snowball sampling, where enrolled participants recommended additional key informants from within their social, professional and family networks [30]. NR made initial contact with potential participants to gauge their interest in participating in a one-hour research yarn. Interested individuals were sent the study information and had the opportunity to ask questions before providing verbal informed consent to participate.

### Data collection

**Fig. 1:**
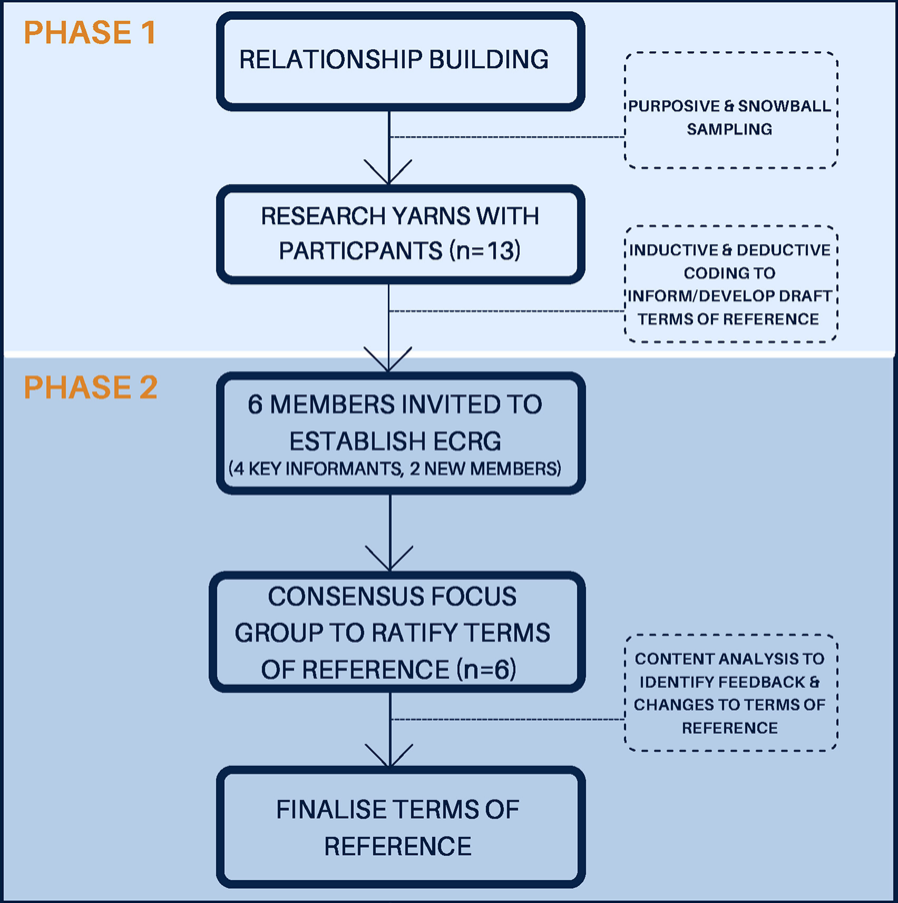
Overarching methods

Data were collected in two phases. Phase one involved semi-structured research yarns which aimed to explore stakeholder perspectives of establishing a representative Aboriginal community reference group and to inform the development of a draft terms of reference. Phase two involved a culturally secure consensus focus group with the established community reference group to ratify the terms of reference [31] (see Fig. 1). In phase one, data were collected by PO or PO and NR collaboratively, through semi-structured yarning interviews with participants between March 2020 and November 2020. Participants who expressed an interest in participating were offered the option of face-to-face, phone or teleconferencing yarns at a time convenient to them, however due to COVID-19 restrictions at the time of data collection, only two face-to-face research yarns were able to be conducted. While all participants were offered the option of having an Aboriginal co-investigator present at their interview, three participants declined this offer due to having an existing professional relationship with the first author, signaling that they felt comfortable to proceed one-to-one. The yarning schedule was designed in collaboration between Aboriginal and non-Aboriginal members of the research team and was guided by the NHMRC Model Framework on Community and Consumer Involvement in Research [14] as well as domains commonly covered in terms of reference documents (purpose, authority, decision making, roles and responsibilities, governance and function). These domains were also informed by examples of terms of reference for Aboriginal reference groups previously established by senior Aboriginal researchers within the ECCO collaboration (see example  semi-structured yarning schedule in Additional file 1). Each research yarn commenced with a social yarn, enabling trust to be fostered with each participant before moving into the research yarn [28].  Research yarns lasted between 35 and 90 min, with an average length of 53 min. All participants were given the opportunity to review their transcript upon their request to check that it accurately reflected their experiences. One participant reviewed their transcript before approving it to be included in the final sample.

In phase two, we used recommendations identified from participant research yarns about the composition of a representative community reference group to invite six Aboriginal people to become members of the ECCO community reference group (ECRG). Four out of six invited members participated in phase one of this study. All invited members consented to join the group and to participate in a culturally secure focus group during the first ECRG meeting. The purpose of the focus group was to come to agreement on, and formalize, the draft terms of reference document developed in phase one of this study. RP and PO both facilitated the focus group. The focus group was adapted from the consensus group technique outlined by List et al. [31], however we embedded strategies to enhance the cultural security of this process. Examples of these strategies included; allowing time for social yarning prior to and at the conclusion of the meeting, including an extended Acknowledgement of Country (see Box 2) and ensuring male and female Aboriginal co-investigator (NR and RP) were present and assisting or leading the group. We also emphasized the self-determination of the group. For example, being transparent about the process of the focus group whilst being flexible and reflexive to the input of group members and implementing ongoing changes to meet the needs of the group as they arose. Members were encouraged to attend face-to-face in a private and safe meeting room to allow the members to meet, socialize and build connections with one another. Light refreshments were provided from a local Aboriginal business and each member was reimbursed with a Visa gift card for their time. Following a brief social yarn and introductions, each domain or section heading of the draft terms of reference was read aloud for members to comment and provide feedback on. A process of tacit consent (i.e. implied agreement without being vocally stated) and vocal dissent was used to streamline this process. Any issues, feedback or suggestions raised by members were discussed as a group. Disagreement and dissent were managed by group negotiation, whereby any changes to be made to the terms of reference or function of the ECRG were adjusted until all members were happy to proceed. Pen and paper were also provided to allow members to convey issues in confidence if preferred. The formal section of the focus group ran for 1 h and 15 min, with social yarning continuing after the voice recorder was turned off. In the week after convening the focus group, members were followed up via phone by NR and encouraged to provide additional feedback on the process of the meeting. Research yarns and the focus group were recorded, transcribed verbatim by either PO or an external transcription service.

### Data analysis

Data in phase one were analyzed in parallel with data collection using a modified framework approach [32, 33]. Framework analysis is a flexible qualitative analysis method used to sift, chart and organize data in accordance with key issues or themes [32–35]. In step one, one author (PO) became familiar with the transcripts through the transcription and editing process and by re-reading the transcripts. Transcripts were then uploaded into NVivo (QSR International Pty Ltd. Version 12). In step two, two researchers (PO and RP) coded the transcripts line by line using open coding to identify broad concepts relating to meaningful community engagement in the context of the ECCO research program as well as concepts related to establishing a community reference group. In step three an analytical framework was developed using pre-determined domains relating to developing a terms of reference document as well as concepts identified in step two. The pre-determined domains included; purpose, authority, decision making, roles and responsibilities, governance and function. Concepts identified in step two and included in the analytical framework were; recruitment, size of group, representation, reimbursement and engagement. In step four, one author (PO) applied the analytical framework to all transcripts charting the data into a framework matrix using Microsoft Excel. The framework matrix consisted of one row per participant and one column for each of the analytical framework domains. Using deductive coding [36], important concepts from within each transcript were extracted and mapped to the corresponding participant/domain in the matrix. Three authors (NR, RP and a third Aboriginal PhD student with a background in physiotherapy) applied the analytical framework to a subsection of the transcripts. The charting process allowed the research team to gain a better understanding of important concepts between and within each participant whilst noting similarities and differences. In step five, the data were further summarized and collapsed by describing the main categories within three overarching themes. In step six, the draft terms of reference were developed. The overarching themes, categories and analytical framework informed the section headings of the draft terms of reference. The draft terms of reference were presented to the authorship team comprised of both senior Aboriginal and non-Aboriginal researchers to allow components of the document to be refined and challenged.

In phase two, RP reviewed the focus group transcript to identify consensus outcomes in the focus group data. First, RP read through the focus group transcript to identify any feedback or recommendations that ECRG members raised about the draft terms of reference. For each suggestion identified in the focus group transcript, the main points of discussion and consensus outcomes were recorded. A consensus outcome was defined as the point at which all members agreed upon a change to be made to the draft terms of reference or function of the ECRG. These changes were then incorporated into an updated version of the terms of reference by RP and PO and distributed to the ECRG members to confirm that the changes made accurately represented their input.

## Results

The final sample in phase one included 13 participants (eight female and four male), 12 who identified as Aboriginal and one whom identified as Aboriginal and Torres Strait Islander. One invited participant formally declined due to a lack of time. Participants represented a range of ages. While all were residing in Victoria, Aboriginal participants also identified as Wurundjeri, Nimanburru, Wiradjuri, Yamatji, Narrunga-Kaurna, Gooreng Gooreng, Kamillaroi, Wuthathi, Gunditjmara, Wotjobaluk, Ngarrindjeri and Taungurung and one Aboriginal and Torres Strait Islander participant identified as Mabuiag. Three out of 13 participants were considered Elders in their community. Participants came from diverse professional backgrounds including physiotherapy, nursing, general practice, health services management, hospital liaison, cultural safety education, health and medical research and the arts, and represented organizations such as Universities, Aboriginal health research organizations, Aboriginal Community Controlled Health Organizations (ACCHOs) and hospitals. Seven participants also identified as identified as having either lived experienced of osteoarthritis. These individuals experienced osteoarthritis or total joint replacement surgery, or were currently living with a family member who experienced osteoarthritis. Ten participants had previous experience as members of advisory committees or reference groups.

**Fig. 2:**
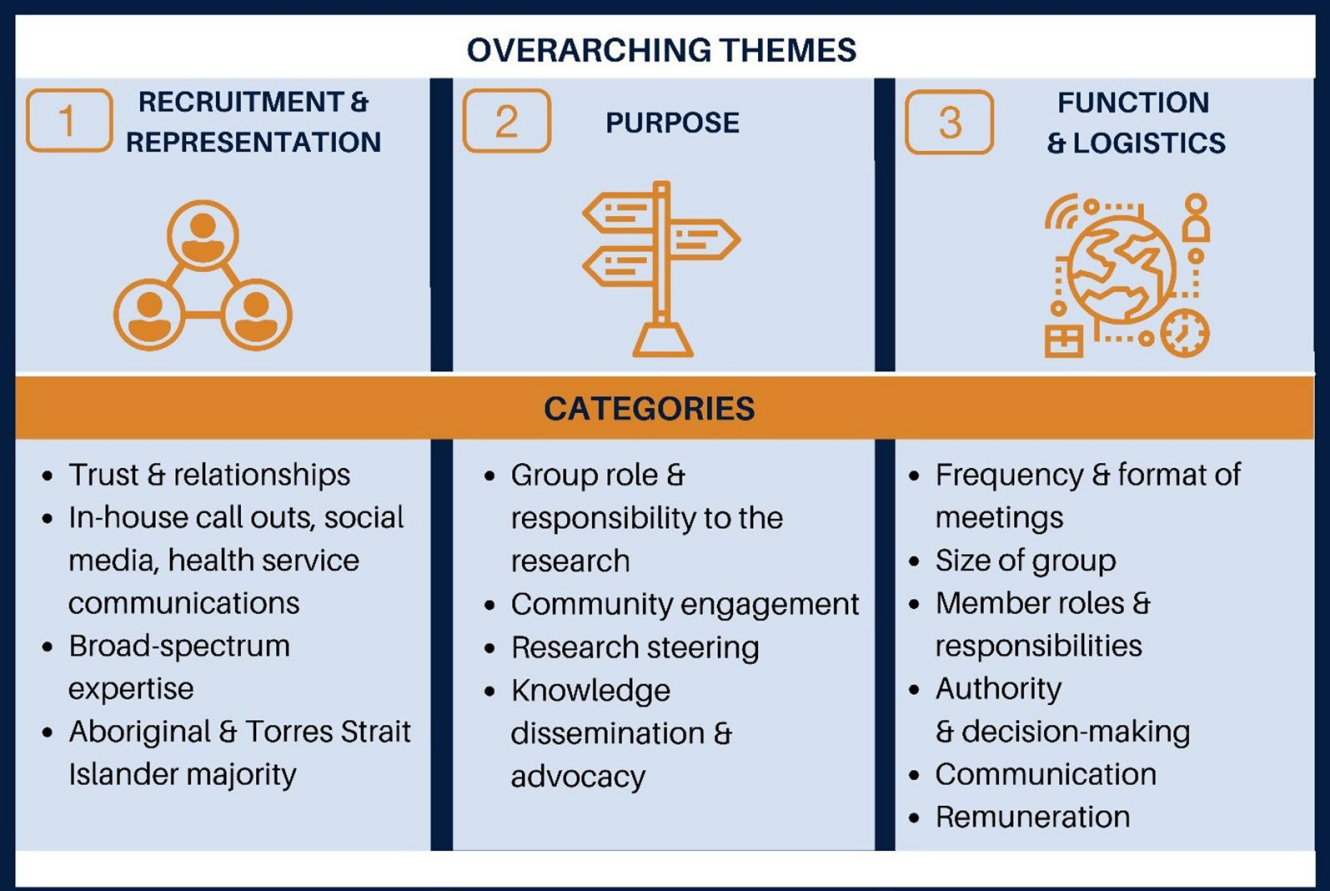
Overarching themes and categories identified in phase one interviews

Results from phase one are presented in four tables which represent the three overarching themes identified (Fig. 2): Recruitment and Representation, Purpose, and Function and Logistics . Within each table we highlight supporting quotes for each category mapped to these themes as well as citing where we incorporated these recommendations in the draft terms of reference (Additional file 2). Supporting quotes are indexed by the participant identification number and their gender (e.g. Participant 1, F) with further participant characteristics being omitted due to the specific nature of our participant group and the risk of these features allowing participants to be identified.

We used the findings of phase one to invite six Aboriginal people to become members of the ECRG. Four out of six invited members were participants who we had interviewed in phase one of this study. An additional Aboriginal female with lived experience osteoarthritis and a manager of an Aboriginal health service were identified through networks of the research team. The final six ECRG members (four female, two male) had lived experience of osteoarthritis and joint replacement surgery, a family member of someone who experiences osteoarthritis, a male Elder, a senior Aboriginal health researcher, a physiotherapist and representatives from both regional and metropolitan health services.

### Phase 1: Key informant yarning interviews—developing a draft terms of reference

Three themes were identified in the phase one interviews. These were Recruitment and Representation (Tables 1, 2); Purpose (Table 3); Function and Logistics (Table 4). These themes and the categories identified within them are represented in Fig. 2 and each of the three tables below.

**Table 1:** THEME 1—Recruitment and Representation

Recruitment and Representation	Suggestions identified from interviews	Supporting quotes	Reference in draft terms of reference
Trust and relationships	When reflecting on successful recruitment of ‘the right people’ to a community reference group, the main theme identified by participants was the importance of relationships. Five participants referred to the importance of building trust or relationships for recruitment purposes. Many also referred to community leaders, Elders or ‘gatekeepers’ of community serving critical roles in assisting researchers in forming the networks needed to facilitate member recruitment.	“*You need to build a relationship with people and you need to find your gatekeepers. If you haven’t got a relationship with the community, gatekeepers will open the way”* – Participant 12, F *“Try and link in with one or two community leader-type people that know a lot of people. That’s how you’ll get them. Forget formal”* – Participant 2, F *“Be transparent with community, so you can build relationships and build trust, which is key"* – Participant 6, F	N/A (related to recruitment rather than terms of reference)
In-house call outs, social media, health service communications	Secondary to relationship building, the use of strategies such as social media, community notice boards and the communication channels of relevant health organizations (e.g. newsletters) were also suggested by some participants. Despite these ‘in-house call outs’ being more trustworthy, one participant cautioned about these potentially cursory strategies, as these strategies were less focused on relationships, may lead to members of the group being less committed and engaged in the group over time.	*“Reach out to Aboriginal organizations to do a callout, that way recruitment for the reference group is in-house within community*” – Participant 6, F *“A poster on a board in an Aboriginal Community Controlled Health Organisation just does not crack it, you’ve got to have a relationship with them to ask them to come on board”* – Participant 12, F *“Social media [is] big as well. I got a lot of people who I didn’t expect from social media ads on different black fella groups” –* Participant 2, F	N/A related to recruitment rather than terms of reference)
Broad-spectrum expertise and Aboriginal medical services	Through the above recruitment strategies, most participants recommended representatives of a broad spectrum of expertise around the table inclusive of ‘both sides of health services’ or both service providers and consumers. Most participants highlighted the importance of engaging Aboriginal health services either through the state ACCHOs (see Box 2) or through engaging with individual community or medical services (see also Table 2).	*“It's very hard, especially when you're engaging clinical staff and people from a research background who have this wealth of knowledge, but then you also have that lapse and lack of cultural knowledge, and then you have the community who may or may not have a vast clinical background in it but have those lived experiences or have that knowledge of what osteoarthritis is at a base level. Being able to balance the two out I think is important”*—Participant 1, M *“I suppose in locations where you’re looking to do the research, I think certainly the Aboriginal medical services, should be really the first people to be engaged, primarily because they’re the ones who would be dealing most with Aboriginal patients. Yeah. I would certainly suggest that they be involved. Whether that’s through the individual services themselves, or through VACCHO”* – Participant 11, M	Additional file 2, Page 5 under section ‘Membership’
Aboriginal and non-Aboriginal representation	Despite the overlap of expertise and demographics to be represented on the group, there were divergent views around the inclusion of non-Aboriginal people as members of the ECRG. One participant referred to racism and the ongoing marginalization of Aboriginal people in the design of health services. Two participants felt it was important to include non-Aboriginal people as allies on the journey towards better health outcomes. Whilst exploring these themes, a few participants offered up solutions whereby non-Aboriginal people could be included in positions that could not be filled by Aboriginal people or having a set proportion of the group that must be Aboriginal, especially when considering reaching quorum. This predetermined proportion was to be maintained with any members turnover and if proxy members were to be filling in as sitting members.	“*Do you think that when they’re designing stuff for white people they ask Aboriginal people to be on [the group]?”*—Participant 3, F “*It’s really important on any Aboriginal project, we involve as many Aboriginal people as we can, but I feel like if we’re going to be working towards reconciliation, health, closing the gap, we need to be working with our allies towards this. It’s going to open people’s eyes to how big this gap is, how important Aboriginal health is and it helps spread the message” –* Participant 9, M *“Involving non-Aboriginal members as well in the group is important, having non-Aboriginal researchers, with a background around Aboriginal research would be great”* – Participant 1, M “*I would say a specific percentage of your group must be Aboriginal or Torres Strait Islander. So, 80 per cent Aboriginal or Torres Strait Islander, 20 per cent not. You need logistics around it”* – Participant 9, M	Additional file 2, Page 5 under ‘Members’

**Table 2:** THEME 1—Recruitment and Representation, suggested members to be recruited to the ECRG

Suggested representation	Key informant												
	1	2	3	4	5	6	7	8	9	10	11	12	13
Aboriginal Elders	✓	✓	✓			✓	✓	✓		✓		✓	
Lived experience of osteoarthritis	✓	✓	✓		✓	✓	✓		✓				
People who live with someone with osteoarthritis	✓	✓	✓						✓				
Aboriginal medical services or ACCHOs*	✓			✓	✓		✓	✓	✓	✓	✓		✓
Aboriginal health workers/nurses/clinicians	✓	✓	✓	✓	✓	✓					✓		✓
Hospital-based Aboriginal health units or Aboriginal Liaison Officers	✓	✓				✓		✓			✓		✓
Aboriginal academics/researchers		✓	✓			✓		✓	✓		✓		
Geographical representation		✓						✓					
Younger people/age diversity	✓	✓			✓	✓							
Gender diversity					✓								
Non-Aboriginal representative	✓		✓						✓				

^*^Aboriginal Community Controlled Health Organisations (see Box 2)

**Table 3:** THEME 2—Purpose

Purpose	Suggestions identified from interviews	Supporting quotes	Reference in draft terms of reference
Overall purpose	Participants described the main overarching purpose of establishing a community reference group as being a mechanism to steer, direct or navigate the research through a cultural lens. Many participants stressed the importance of the group’s purpose being beyond that of ‘cultural business’ or exclusively for cultural guidance, highlighting the breadth of expertise that the members would inevitably bring to the table. Some participants also described the role that power plays within research and health settings and the purpose of the community reference group in bridging that gap for community members. By bringing together Aboriginal people with diverse professional and lived experience to provide research steering, and by giving Aboriginal community members a voice, the overarching purpose should also be to improve the health outcomes of Aboriginal people.	*“If you’re doing work with Aboriginal people, you need to take Aboriginal people along the way”* – Participant 6, F *“I think [we’re] your navigators, not just in cultural business but in so many other areas… we can be seen as just catering to all things Aboriginal, but there is so much that a reference group can offer beyond that—amazing researchers that have come up against adversities…I always like to think that I’m not just being used because I’m Aboriginal. I bring a wealth of knowledge; administration, project planning, budgets that I can bring to that group”* – Participant 2, F “*There is a power gap between, a clinician or researcher and your average community member. Aboriginal people, we're aware of that power gap, and in a lot of instances we don't feel comfortable voicing our opinions, so bridging that power gap [with the group] is important”* – Participant 1, M	Additional file 2, Page 3 under ‘Purpose’
Group role and responsibility to the research project	Many participants highlighted the role of self-determination and the need for the group to have ownership of its own role and responsibility to the research project. One participant echoed this strongly, highlighting that the proposed terms of reference should only be a draft to be shaped as they see fit. Although the group should decide upon their own role and responsibility to the research group, which would ultimately be determined by the capacity of the group, participants provided working suggestions which included facilitating community engagement, research steering, knowledge communication, dissemination and advocacy.	*“You need to ask them what the group wants to achieve in terms of their responsibility for the overall research project. They need to set that up themselves from the start. Does that mean they only provide guidance on cultural protocols for data collection, or does it mean they have input to every different step in the process?”* – Participant 2, F “*You need to be transparent that what you propose to them is a draft, and they can shape it how they like*” – Participant 11, M	Additional file 2, Page 3 under ‘Role’ and page 5 under ‘Authority’
Overarching aim 1: Community engagement	Most participants acknowledged the role that community engagement would play, with the concept of ‘giving voice’ to community often being cited. A few participants also highlighted that community engagement efforts should go beyond that of ‘consultation’ with ongoing involvement of community reference group members and community members, rather than just ‘a chat’ at the beginning of the project. Community engagement was vital to ‘ensure that the engagement of the Aboriginal community is not 'taken for granted’ and to ‘build an authenticity’ with community which the research group aims to benefit and should be done in a way to avoid tokenistic or ‘check box’ gestures.	“*You’ve got the right people to take the opinion of the community and make sure it’s heard*” – Participant 8, F *“I think to make sure that the engagement the Aboriginal community is not taken for granted. That when the group, as a representative body of the community, provides advice to the research project that it’s actually that of the community”* – Participant 11, M *“I guess the plan obviously is just involving people in the discussions, and hearing people’s voices from the ground roots. So, even times when I’m talking at the things I’ve been involved in, I can kind of share the stories of people that I’ve seen, but it’s better to actually get them involved”* – Participant 13, F *“Well, there’s consultation and then there is what’s needed. I think sometimes when research is done, consultation is used as a check box. I think you need to be very aware that a lot of community members are really wary of consultation, because often it takes knowledge from communities, uses knowledge for the benefit of whoever institutions or researchers or organizations and the people that have given their time and effort and their knowledge lose it, essentially”* – Participant 2, F	Additional file 2, Page 3 under ‘Role—Community Engagement’
Overarching aim 2: Research steering	Research steering and related aims were the most commonly suggested by participants, which closely aligned with the suggestions for the overall purpose of the group. Cultural security and the group providing a cultural lens was also discussed in the context of research. With regards to steering some participants suggested that the community reference group should have input in the research at every step of the way. One participant suggested methodological input for qualitative studies at a minimum.	*“To steer, say, the research, and whether it's the findings or it's the way the research is conducted, in a culturally safe manner. We need to ensure that community members being engaged with feel safe, and the only way to do that is to engage the community themselves. I know it's a lot of repetition, but we know our community, we know how it works, we know people who would be easier to engage with than others. We know, I guess, the right way to talk, we know how to build that trust with that patient quickly and in a way that would be beneficial to research”* – Participant 1, M *“Absolutely you navigate [the research]. We’re going to see things that – and the people that are standing with different lenses and just seeing the same things that you wouldn’t necessarily see”* – Participant 3, F	Additional file 2, Page 3 under ‘Role—Research Steering’
Overarching aim 3: Knowledge dissemination and advocacy	A few participants acknowledged the role that the community reference group should have in being able to communicate research findings, project outcomes or project needs to community or community health organizations. Having a strategy for communicating with community was seen by a few participants as an important step to ensure that research was being translated into practical outcomes—an ethical requirement of Aboriginal health research.	*“You might want to ask yourself, what influence do those Aboriginal people that come onto your committee have back in their community or their entities, and how can there be a two-way information flow? So, your committee can share back, whether it’s to the community cooperative health and welfare organization”* – Participant 7, M *“Also, I think this is probably nearly the most important one, is around the translation of the outcomes from the project into actual practice*” – Participant 11, M “*The other thing they absolutely are is your comms strategy – your communication strategy. If you want to get anything out there or you want to get anything back from community they’re the people that are going to do that for you”* – Participant 3, F	Additional file 2, Page 3 under ‘Role—knowledge dissemination and advocacy

**Table 4:** THEME 3*—*Function and Logistics

Function and logistics	Suggestions identified from interviews	Supporting quotes	Reference in draft terms of reference
Frequency	Many participants suggested that the frequency of meetings would be determined by the ‘cadence’ of the project, which included both the intensity of the work that needed to be completed and the timelines of the project. Frequency of meetings were also suggested to be decided on by the group as this would ultimately be decided by the capacity of the members. Specific time frames suggested for meetings ranged from a maximum of fortnightly to six-monthly at a minimum. Many participants suggested that meetings should be more frequent when the group is first established and should move to less frequent as the group becomes more familiar.	*“It depends on what the timeline of the project is. If you’ve got tight timeframes, monthly. If you haven’t got tight timeframes, bi-monthly is really good.*” – Participant 3, F “*I would probably say once a month at the start, and then move into bi-monthly and then quarterly*.” – Participant 1, M *“In the first instance you’d probably want to do it once every couple of months; but really I’d be asking the group that question once you’ve formed it. When you’re seeking people’s interest in being part of the group, they can say, “Okay, well, I understand the commitment in the first sense,” so, “over the first six months we’re going to meet three times,” but then they’ll develop a terms of reference, which will [state] how often they’ll meet*” – Participant 11, M	Additional file 2, page 7
Format of meetings	Most participants acknowledged the importance of having meetings available through teleconferencing platforms such as Zoom; this was to improve the accessibility of meetings and to lessen the burden of members that would inevitably be busy. Access to the internet and software such as Zoom was highlighted as something to consider among older community members. Some participants highlighted the importance of meeting face to face for the first meeting to allow for social yarning, meeting and building relationships between the group.	*“I think the first time if you can bring people together, do it that way, when everyone first meets each other”* – Participant 3, F *“I think—given our time—Zoom’s definitely something that we kind of need to use. But again, a lot of things that we do, and some of the working reference groups that I sit on for work, we do have Zoom catch-ups, but I think accessibility is something that you just need to be mindful of, especially with logistics and things like that; so how are community members, particularly older community members, going to access it?”* – Participant 6, F *“In the current environment, yes. We’re kind of forced to use [Zoom], and I think it’s the safest and the actual best way. Not just for cultural appropriateness, but for the whole of Victoria to use this. I think it’s really important that the people that are coming onto the group know how to use this as a platform…[and] when we can meet I would prefer to meet in person. The time of people is so much valuable, but still offer a dial-in, a dial-in teleconference number or teams to dial in people who can’t be there”* – Participant 9, M *“But I think it’s really important to have the chair the first time – like I said, if you can meet face to face it’s really important to do it and always do your Acknowledgement of Country. I know you guys know that but, yeah. That always is really good. Then making sure that everyone goes round and they say which country they’re on. So taking that time to do that.”* – Participant 1, M	Additional file 2, page 8
Size of the group	Suggestions for the ideal size of a community reference group ranged from a minimum of four and a maximum of 12. Many participants discussed potential challenges of logistics, availability, absenteeism and overall management of a group that was larger than 12. Some participants also suggested that the group should be large enough to allow for absenteeism while still remaining large enough to make decisions.	*“As a caution is you may not get as many people as you would like but the people you get are the right people”* – Participant 12, F *“More than four, and probably less than 10.. I think if you’ve got, three or less you’re not really getting a particularly good breadth of discussion across the group, and I think unfortunately there’s an opportunity to be loaded one side or another when there’s not enough people. Then anywhere up to 10’s fairly easily managed, but above that it’s just too hard, you’ve just got too many people and too many things that they want to say. Nightmare… just the logistics and being able to coordinate that many would be hard.”* – Participant 11, M *“Again, I think sending an agent when someone cannot attend who has been well informed is important”* – Participant 1, M	Additional file 2, page 5
Roles and responsibilities	Appointing a chair or co-chairs to provide governance to the ECRG was considered essential to nine participants. Participants saw the chair as someone who needed to be a good facilitator, someone who could ‘keep track of everything’, follow up on peoples’ responsibilities and accountabilities, keep people engaged along the way, as well as someone who was Aboriginal and who potentially had a good grasp of Aboriginal health research. The chair or co-chairs were seen as necessary to provide a ‘point person’ for other members to communicate with. One participant suggested that both a male and female leadership should be instated. Additional specific roles suggested were a community liaison officer (to communicate with Aboriginal health services and community members) and an admin/secretariat support person. Roles beyond that of the chair were more often described as ‘small goals and responsibilities’ or ‘something to be involved with or be in charge of’ that would provide each member with a sense of shared accountability. Members roles were also seen as something that the group should have ownership over.	*“I think it would be good to have someone who can perhaps be a chair of the group that has a good grasp of Aboriginal health research. They don’t necessarily have a full understanding of this particular project, or a PI on this project or anything, but understands the research process particularly in an Aboriginal context, have got a good understanding of how to engage with Aboriginal communities in health research, are able to understand good governance of these kinds of things, and perhaps find someone like that that can really manage the group”* – Participant 11, M "*Community should come up with roles that they want to play, and what their expectations are for every single person in that…it should be done in a way where they agree with doing that, because they came up with it”* – Participant 6, F *“Sometimes they help, but I think they aren’t essential, I would get the group together first and see how it gels and see what people want to do”* – Participant 5, M *“You need to give people little responsibilities and somebody who is responsible for following up that those things have been done basically as the one who is keeping track of everything”* – Participant 10, F	Additional file 2, page 6
Authority and decision-making	The authority and decision-making power of the group in relation to their influence on the research project was an important theme to most participants. Overall, the authority of the group would be an important mechanism to ensure power-sharing and to ensure that the ECRG was meaningful and valued. Transparency around the power of the ECRG from its inception was integral to moving past being considered a ‘token-gesture’ or the group being done ‘just for the sake of doing it’ and was the key to members feeling they were involved in something that has ‘integrity, impact and value’. Two participants saw the ECRG’s authority as more of a ‘checks and balances’ responsibility, rather than the group having the ‘final word’. Participants most often spoke about decision-making in relation to the power of the group and its role in making decisions for the research group and did not offer suggestions for implementing formal, internal decision-making processes.	“*What power will the group actually have, do they have the ability to veto certain elements of the project, who has ultimate say on what the project does? I think if you’re going to go down the path of seeking a community reference group, you need to ensure that their voice is heard and respected throughout that whole process, rather than it just being a token gesture*”- Participant 11, F *It’s good to put out clearly what they are there for. Is it to make decisions? Is it to say yes and no to the way things are going or is it just a sounding board. I think you’ve got to be bold enough to say the reference group has no decision-making powers, but ‘this’ is what you want them to do”* – Participant 5, M *“I think that [the research] should just be double checked with anyone that’s on the committee, if someone isn’t happy with it, then you make a priority of sorting it out. So, it’s not that they’re making all the decisions, it’s just making sure that everyone is happy”* – Participant 8, F “*There is never going to be a final word, there will never be consensus, but you will be able to get an indication more so of stopping things if things are going wrong*” – Participant 2, F “*What constitutes a quorum? You don't want to have two or three people show up and decisions being made when they shouldn't be made, when most of the group representation isn't there. You want representation for those decisions”* – Participant 1, M	Additional file 2, page 5, and page 7
Communication	Some participants provided suggestions on communication strategies which may help to keep members engaged over time. A few participants suggested that a centralised location such as within a social media platform (i.e. a Facebook group) may help to keep communication less formal and more continuous than emails.	*“Formal does not work very well. If you’re stuck with emails, it just gets way too confusing, too many reply all threads going all over the place. Having a centralised location where you can have continuous communication is really handy. And then for those bigger pieces, you can use email” –* Participant 2, F *“I think just touching base every now and then and making sure that everyone’s still happy and still onboard and still have the same opinion I guess or the same direction. I guess sometimes you change your mind or things change along the way. I think it’s constant checking in is always good” –* Participant 8, F *You hope that everyone will stay engaged…I think if you guys have frequent comms [communication], but not overly overbearing, making sure that everyone is replying, and maybe reaching out to everyone and saying, ‘How’s this going?’ This is what I mean by that group owner keeping people connected. It’s not like pushing and being like ‘How’s your piece of work going?’ It might be like ‘How are you going? How are you liking being on the committee?" –* Participant 9, M	Additional file 2, page 7, under section ‘Expectations’
Remuneration	Most participants agreed that members should be reimbursed for out of pocket costs if attending meetings in person. Participants had differing opinions about sitting fees and the appropriate amount that members should be reimbursed, however for those that suggested financial reimbursement there was a sense that this would help to ensure members felt valued. Some participants felt that it was appropriate to only provide refreshments or to provide a gift instead of monetary remuneration, however others felt that it was important that members felt valued and recommended a range of sitting fees between $50 and $200* per hour or meeting.	*"If you want the mob and you just serve up cold sandwiches on a winter’s day, you're not going to get much" –* Participant 5, M *“Most [reference groups] I have been on paid a stipend for each meeting. I think that’s the standard…and that they get reimbursed for any travel, time they’re attending the meeting. And then if there’s work to be done outside of the meeting, I would consider reimbursement for that as well”* – Participant 2, F *“It depends on how much time you’re expecting that person to commit. So, not just the meeting, but what work they’ve had to do as well. Yeah, I don’t know, it’s tricky. Let’s just say your hourly rate is like $50, but you’ve had to do a couple of hours work beforehand, you might be looking at 150 or $200 or something per meeting”* – Participant 13, F *“It doesn’t have to be big but it’s substantial to culture and local businesses ‘hey, we’re going to give you a little gift that includes this, this and this from these local [Aboriginal] businesses”* – Participant 9, M	Additional file 2, page 8 under section ‘Sitting fees’

^*^The research team discussed remuneration further with senior Aboriginal members of the research team who felt that $100 (+ refreshments and any travel re-imbursements) advised this was appropriate and consistent with similar activities in other areas of health research. This figure was also agreed upon by all final members of the ECRG upon before their appointment to the group

### Phase 2: Consensus focus group—formalizing the terms of reference

**Fig. 3:**
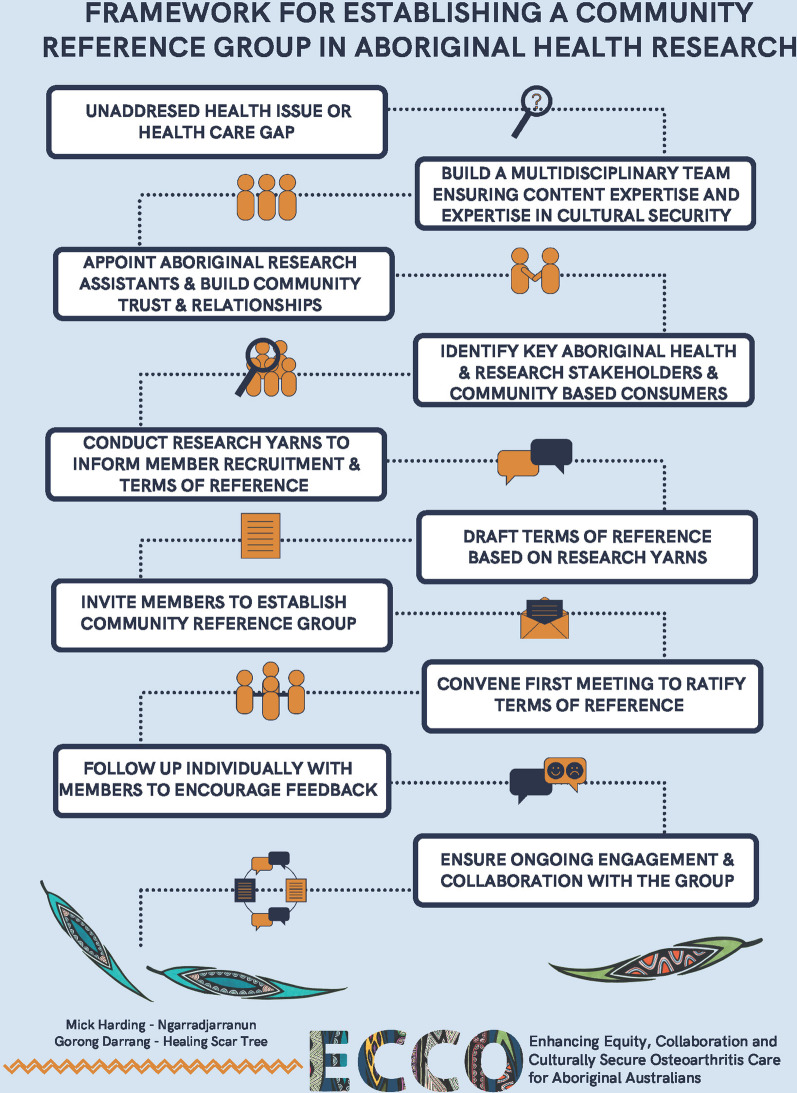
Framework for establishing a community reference group in Aboriginal health research

Based on the key informant recommendations in phase one, six people from within established community networks were invited to become members of the ECRG and ratify the draft terms of reference were (Additional file 2). A summary of the changes made to the draft terms of reference through the focus group are outlined in Table 5. Following the focus group, participants were invited to review the final terms of reference to check that their recommendations had been accurately incorporated into document. This process revealed that individual follow-ups with ECRG members also provided us with valuable, constructive feedback on this process (as stated in Fig. 3) which was also used to further develop the terms of reference. The final version of the terms of reference is provided in Additional file 3.

**Table 5:** Changes made to draft terms of reference through the focus group

Terms of reference section	Concerns raised by group members	Supporting quote	Changes made through consensus
Definitions	A member of the group raised a concern about the use and origins of the term ‘cultural security’, with preference for the use of the term ‘cultural safety’.	*“What I mentioned was that cultural security doesn't come from an Aboriginal person. It was born out of bureaucracy and bureaucracy labeling what they were doing. Cultural safety comes from a Maori nurse. So it takes in the paradigm of colonization and racism which is core to its processes, cultural security, however, the majority of the time people are talking about it, it makes sense because its born out of bureaucracy, it doesn't talk about colonization or racism” –* Participant 3, F	The group discussed both definitions of cultural security and cultural safety and agreed that both were required to describe different situations or contexts. Definitions of cultural safety and cultural security were differentiated at the outset of the document. References for both definitions were provided from Indigenous academics who have been pioneered this work (Coffin, Ramsden). ‘Research Steering’ aims of the ECRG were amended to separate advice on cultural safety and cultural security and for advice of all forms of ethical conduct.
Role (aims and responsibilities of the ECRG)	A member of the group was uncomfortable with the notion of representing a whole organization or whole community.	*“I'm probably just as an individual and when I read through this, I'm not comfortable representing a community. I think maybe if you want to just set individual families, countries that don't represent the community or represent myself, my views” –* Participant 3, F	The ‘Community Engagement’ aims of the ECRG were amended to reflect that members do not represent entire communities, that they are individuals from their respective communities who provide advice for, not on behalf of Victorian Aboriginal communities.
Authority	Members of the group requested additional information about their authority and decision-making power in relation to the research group. With this, group members requested more information about where the ECRG sat in terms of the wider research group and how communication would be facilitated between the ECRG and ECCO researchers.	*“Say for instance we make a particular decision on something here and it goes up the chain to the other group, and if it doesn't get used, whatever the recommendations is, the decision we make, how does it get communicated back to us about why they might not use that that recommendation?” –* Participant 7, M	The group discussed that ultimate decision making would rest with the research group, however advice from the group would be prioritized To highlight the above, the ‘Authority’ section was amended include that all ECRG advisory and recommendation outcomes will be communicated back to the group in a transparent and timely way. An organizational chart was added to help members visualize where the ECRG group sits in relation to the research group, other reference groups and how these might interact and to assist with transparency of how information will be shared among the group.
Membership—Proxy	The group discussed what characteristics a proxy should have to sit at an ECRG meeting. A member of the group requested that there be clarification on the decision-making power of proxy members and if they were able to vote on tabled items and contribute to a quorum.	*“When we're arranging meetings, do proxies have to have certain characteristics?”*–Participant 15, F *“Do proxies have decision making rights?” –* Participant 14, M *“[without decision making powers proxies] are likely going to slow you down in whatever you're doing down…I reckon, the key to this operation, the communication, behind it. How you made the decision why you made the decision” –* Participant 7, M	The group discussed and reiterated what was stated in the draft terms of reference: that proxies should have similar characteristics to the member that they are representing. The proxy should be Aboriginal if the member is Aboriginal and should represent a similar profile in terms of professional or lived experience. Guidelines were added stating that proxy members are allowed to vote on items if given authority by their respective member.
Meeting process—decision making	Members raised that using percentages to define majority or quorum was too difficult to work with and suggested half + one as an easier alternative that worked for many members in the past.	*“I'm used to half plus one. That's what I'm used to, so it makes it because.... Once you start getting the 75 to 80 percent, it's more like constitutional type thinking, which is which is a really high bar compared to a simple majority plus one”* – Participant 7, M *“Yeah, I'm used to community forums and in governance forums, for Aboriginal and Torres Strait Islander people that you just talk it out till you get to a point that you're happy with” –*Participant 3, F	The terms of consensus/quorum were changed. Should a vote be required, majority was changed from ‘ > 80%’ and defined as ‘half + one’.
Meeting process—documents	A suggestion was made to add ‘Sorry Business*’ as a standard agenda item after the Acknowledgement of Country. This would allow space to pay recognition or allow a minute silence for any community members who may have passed away. A member also suggested that naming it officially as ‘Sorry Business’ may be too sensitive and not appropriate in some communities or situations. A suggestion was made to include both a check in and Sorry Business as this agenda item to allow space for any members to voice any personal items they feel comfortable to.	*“In loads of other meetings I attend we have Sorry Business on the table and so if people are comfortable to put those names forward and stand and have a moments silence for those who have passed on to the next world. So I think it's a really good to have”* – Participant 7, M *“A lot of the meetings I've been to, we just have a quick check in at the start of the meeting. So not labeled Sorry Business because sometimes that might bring up a lot of sensitivities and feelings into the meeting and then people might be different in that meeting. I'm comfortable with people wanting to raise sorry business, but I think that maybe if we just have a quick check in the start and so people can express anything, not just business and have it focused on that”* – Participant 9, M	The standard agenda format was amended to include both ‘check-in’ and ‘Sorry Business’. A reminder about the potential for sensitivities around any Sorry Business was stipulated. The terms of reference also states that both check ins and Sorry Business will be omitted from the minutes to ensure privacy and sensitivity around any matters discussed.
Expectations	A discussion took place around the most convenient communication channels, document sharing and storage. Members agreed that emails were the best communication avenue but that a more central share point for quick communication and document storage was needed. A Facebook/social media group or newsletters were not seen as important for members.	*“Google Drive or teams or something, because of confidentiality as well…but I think it's probably a good thing to have it in that specific space so that if we need to go back and refer to a particular document at any point in time it’s going to be easy” –* Participant 9, M	A Microsoft Teams group platform was established for data and document storage so that members can easily refer to documents or communicate quickly using the chat function. This platform was decided upon for the chat function, security and accessibility for all members regardless of which organization they were from.

^*^Sorry Business is a term used to described Aboriginal cultural practices associated with death and grieving (see Box 2)

## Discussion

This paper describes the ECCO research groups’ practical experiences in the process of selecting, engaging and developing an Aboriginal community reference group and terms of reference by adopting a PAR approach. Our process and recommendations are described in a 10 step-framework for establishing a community reference group in Aboriginal health research (Fig. 3). We demonstrate how calls for adopting community engagement practices by national research organizations [1, 6, 9, 11, 37] may be appropriately operationalized through establishing a community reference group to provide guidance on Aboriginal health research projects. Findings from this study concur with the literature which highlights that successfully building partnerships or community engagement strategies requires health researchers to work in ways which are collaborative, relationships focused and flexible in responding to the needs and priorities of Aboriginal people [2, 10]. Although we describe key practical recommendations that may assist in this process of establishing a community reference group, our framework (see Fig. 3) does not set out to replace the right of Aboriginal people to choose how Aboriginal health care is designed and delivered. Participants in our study often emphasized the importance of self-determination, ownership and decision-making power in establishing a community reference group. Therefore, our framework should instead be used as a blueprint, starting point or guide for research groups to engage Aboriginal health and research stakeholders and community members to shape as they see fit. For example, research groups seeking to establish a community reference group to guide Indigenous health research globally may input project and context-specific information for each of the 10-steps in this framework. This in turn will allow the community reference group to be informed by the values of the group under study and to be shaped to be health problem or be disease specific. This may be particularly relevant for research groups who wish to implement community engagement practices with Indigenous peoples of countries who share a common history of colonization [2]. Moreover, it should be noted that the process outlined in this study is just the beginning or the entry point of what should be an ongoing commitment to community engagement in Aboriginal health or Indigenous health research. Moving forward from the development and establishment phase, community reference groups should be engaged in an ongoing PAR framework [26, 38]. By establishing a community reference group, Aboriginal community members are able to participate in and provide guidance within the PAR cycle at all levels of the research. The PAR cycle revolves around four main steps: plan, act, observe, and reflect [26]. The ECCO research group aims to adopt this approach by planning each ECRG meeting around the PAR cycle which will ensure ongoing engagement of the ECRG into the future.

Undertaking the task of establishing a community reference group can be complex. Failing to plan for or address potential challenges may lead to weakened partnerships or skepticism about the role of the community reference group [6, 22]. Strategies cited in the literature for overcoming challenges to maintain an effective community reference group include recognising the importance of local Aboriginal knowledge and cultural values and beliefs, becoming familiar with local Aboriginal communities and ensuring that the benefits outweigh the time–cost of participating in such a group [10]. The PAR process we have outlined is both time and resource intensive, a challenge that appears to be common amongst community engagement approaches in Indigenous health research globally [2]. Researchers should not underestimate the time it can take to build meaningful relationships in this context. Having the financial means to employ Aboriginal co-investigators to broker relationships was paramount to the success and a strength of our study as it enabled us to build trust, build on existing relationships and receive cultural guidance throughout the project. In return, we were able to begin to build Aboriginal health research capacity in the field of musculoskeletal health, which was a further strength of the study. Research groups who plan to use this 10-step framework of community engagement should consider assigning research staff to a relationship building role, prioritize building the capabilities of Aboriginal health researchers and plan for these underlying costs when applying for funding. This should in turn demonstrate to funding bodies (and to Aboriginal communities and services) the researchers’ commitment to ongoing community engagement. We also support setting realistic and flexible project timelines. A true commitment to Aboriginal health will require research groups to stretch the boundaries of their usual systems and Western research paradigms to allow for flexible, organic approaches to relationship building and community engagement [2, 6].

Despite these potential challenges, the findings of this study suggest that our PAR approach to establishing a community reference group and terms of reference was effective in building the necessary connections required in the context of the ECCO project. In utilizing culturally secure research methods, for example by harnessing the power of ‘word of mouth’ or snowball sampling, we were able to build trust and relationships throughout our recruitment and data collection journey [27, 30]. These relationships enabled us to formally invite members to join the ECRG, many of whom we had interviewed in the process of conducting phase one of this study. We acknowledge that a limitation of this study was having the pre-conceived idea of establishing a community reference group and that barriers may exist for some Aboriginal community members participating in group-based activities, particularly as they require members to be available at a certain ‘place and time’ [39]. Additional modes of community engagement, for example, by engaging multiple community representatives in one-on-one discussions may enable a broader range of community members to participate in community engagement and involvement activities [39]. Additional methodological considerations were the small sample size in phase one and the use of tacit consent in the focus group in phase two. Although our sample size may be considered small, we believe that data collected through the 13 interviews was rich enough to answer our very practical research aims. We acknowledge that the use of tacit consent in the focus group may have influenced group members willingness to voice their opinions at the time. In the future, each participant could be called upon to speak to each of the focus group items in a round robin process. Despite this limitation, the research team were transparent about the process of the focus group while also being flexible and reflexive to the input of group members and implementing ongoing changes to meet the needs of the group as they arose. We also received valuable, constructive feedback from ECRG members in this process and recommend casual ad-hoc, one-on-one ‘check ins’ (as stated in Fig. 3) to give members an opportunity to privately raise any feedback or concerns and to ensure that members are satisfied with the conditions of their ongoing participation. Being flexible and adapting to the input of the ECRG members’ suggestions was an important modification aimed at enhancing the cultural security of our focus group technique.

## Conclusion

Current literature suggests that Aboriginal community engagement is integral to any health policy, intervention or Aboriginal health research aimed at improving health care. We have described practical strategies that prioritize PAR, including Aboriginal input and voices in every step of establishing a community reference group and terms of reference. The 10-step framework presented may be especially relevant in guiding research groups who seek to explore novel Aboriginal health research areas by building a program of research from the ground up.

## Supplementary Information

**Additional file 1**. Example semi-structured research yarning schedule.

**Additional file 2**. Draft terms of reference.

**Additional file 3**. Final terms of reference.

## Data Availability

Qualitative data generated during this study and reported in this manuscript are stored in audio and electronic written format at The University of Melbourne. Data supporting the findings of this study are available from the corresponding author (PO) upon reasonable request.

## Abbreviations

ACCHO: Aboriginal Community Controlled Health Organisations
ECCO: The Enhancing Equity, Collaboration and Culturally secure Osteoarthritis care for Aboriginal Australians (ECCO) collaboration.
ECRG: ECCO Community Reference Group
NHMRC: The National Health and Medical Research Council.
PAR: Participatory Action Research
