# Supplementary material for: Laying the foundations of community engagement in Aboriginal health research: establishing a community reference group and terms of reference in a novel research field
Source: Res Involv Engagem. 2022 Aug 4;8:40. doi: 10.1186/s40900-022-00365-7 (PMC9354439; doi:10.1186/s40900-022-00365-7)
Supplement: Supplementary file 1 — Additional file 1. Example semi-structured research yarning schedule. [file 40900_2022_365_MOESM1_ESM.docx]

| Introductory questions | VERBAL CONSENT/ QUESTIONS  Tell me a bit about your story? Tell me about yourself?   - Cultural background - Community - Employment history   Have you been involved in research before?  Do you have any other roles in your community?   - Role model - Cultural roles - Volunteering |
| --- | --- |
| Introduction | - Story of ECCO - Community reference group study - First priority for us is to set up a community reference group to guide our study - We have identified you as someone we believe would have valuable information in relation to setting up the community reference group - Additional exploratory questions around ***your knowledge and experience with osteoarthritis*** |
| Community engagement in Aboriginal health research | What is the role of **community engagement** in Aboriginal health research?  Can you share any experiences you have had being involved in any community engagement processes?   - As a researcher - As an Aboriginal person - Experience in committees   **What worked well?**  **What didn’t work well?**  What does cultural security mean to you?  How do you think this is achieved in Aboriginal health research?  In what ways can a research group like us ensure that Aboriginal voices are engaged in every step of our research, from the ground up?   - Ways other than a community reference group? |
| Community Reference Group and Terms of Reference | **What do you think is the purpose of having a community reference group in Aboriginal health research?**   - Function of the group |
| Describe specific project activities for the first 4 phase of ECCO again and big picture | |
| Community reference group continued… | Considering our specific project, who do you think should be represented in the group?   - Why?   What would the main purpose of the group be for our specific research?  How do you think the group should function?   - Number of members - Frequency of meetings - Format of meetings - Governance and decision making - Roles and responsibilities within the group - How to go about recruiting members - How keep participants engaged - Reimbursement or provisions   What are your thoughts on a group like this functioning over Zoom/Teleconference?   - Impact of COVID - Acceptability - Pros/cons   What are terms of reference?   - What would be important to include in our TOR?   What are some of the challenges we might face in setting up and running a group like this?   - How would you overcome these challenges?   Who else do you think we should talk to about setting up this group?   - Specific people you know - People interested in the formal group - Do you know any other community reference groups like this? |
| Osteoarthritis and Aboriginal and Torres Strait Islander people | - **What can you tell me about osteoarthritis?** - **OA/joint pain in Aboriginal people in Victoria? SVHM?** - **Can you tell me about any patients you treat who may experience joint pain?**   - **Draw on specific example or patient story?**   - **Impact – health, social, life** - **Can you draw on any examples of how joint pain or OA may impact patients that you see?** - **Can you tell me about OA in the context of chronic disease?** - What is your understanding of health-related **quality of life?** - What do you think is important to aboriginal people’s quality of life?   As a health professional can you talk to me about how OA should be treated for Aboriginal people?   - What impact do you think OA has on the health-related quality of life of Aboriginal Australians? - What impact do you think OA has on the **physical** well-being of Aboriginal people? - What impact do you think OA has on the **psychological** well-being of Aboriginal people? - What impact do you think OA has on the **social** well-being of Aboriginal people? - What impact do you think OA has on the **spiritual** well-being of Aboriginal Australians? - Do you think there is a need to investigate OA in Aboriginal Victorians?   - - Context of chronic disease     - Need for culturally secure |
| Personal OA experience | - Can you tell me the story of your knee/hip pain?   - - How long? - How is/was your knee/hip pain impacting your life?   - - What things were the hardest for you?     - Physical/social/spiritual - How do you cope with your knee/pain? - Where did/do you go to get help? - Tell me about what happened when you went to X service?   - - Were there any problems finding care your knee/hip pain?     - Can you describe the care that you got? - How did you come to call your knee/hip pain osteoarthritis? - Are there any things you wished you could access for your knee/hip pain? - Experience of TJR and outcomes - Expectations of tjr? |
| Anything else? | - Gift card! Address |
