# Supplementary material for: Laying the foundations of community engagement in Aboriginal health research: establishing a community reference group and terms of reference in a novel research field
Source: Res Involv Engagem. 2022 Aug 4;8:40. doi: 10.1186/s40900-022-00365-7 (PMC9354439; doi:10.1186/s40900-022-00365-7)
Supplement: Supplementary file 3 — Additional file 3. Final terms of reference. [file 40900_2022_365_MOESM3_ESM.docx]

**ECCO Aboriginal and Torres Strait Islander Community Reference Group**

**DRAFT Terms of Reference**

**Title**

This group will be known as the ECCO Community Reference Group (ECRG).

**Definitions**

**Cultural Safety:** Cultural safety, a concept pioneered by Maori nurse and scholar Irihapeti Ramsden which describes the facilitation of a space and an exchange that is spiritually, socially and emotionally safe, as well as physically safe for people; where there is no assault on, challenge to or denial of their identity, of who they are and what they need. Culturally safe practice requires an understanding of the concepts of culture and racism generally, and acknowledgement of one’s own cultural values and beliefs in particular, in order to avoid cultural imposition. In a relationship where there is institutional power, and historic and ongoing racism, cultural safety is the moment of trust that occurs leading the client/patient/consumer to not needing to protect their difference against the institution or its agents (Ramsden, 1992).

**Cultural Security:** Cultural Security is a concept developed by Aboriginal health researcher and ECCO collaborator, Juli Coffin and describes the embedding of cultural safety into policy frameworks, standard operating procedures and practice. Cultural security is a commitment to conducting research and providing services that do not compromise the cultural safety, rights, values, beliefs, knowledge systems and expectations of Aboriginal and/or Torres Strait Islander people and is the overarching principle that guides the way we do business and create culturally safe environments at ECCO (Coffin, 2007).

**ECCO:** The Enhancing Equity, Collaboration and Culturally secure Osteoarthritis care for Aboriginal Australians (ECCO) collaboration. The ECCO collaboration is an inter-professional team of Aboriginal and non-Aboriginal health practitioners, health service staff, and researchers in Victoria, Queensland and Western Australia who are working together to 1) explore the impact of arthritis on Aboriginal and Torres Strait Islander people and 2) develop a culturally safe and secure model of arthritis care for Aboriginal and Torres Strait Islander people in Australia.

**ECCO Research Team:** Core members of the ECCO Collaboration who are responsible for the daily operations of ECCO research activities in Victoria. The ECRG reports to the ECCO Research Team, who meet for fortnightly progress meetings on Mondays.

**ECRG:** The ECCO Community Reference Group. The ECRG will also be referred to as ‘***the group’*** throughout this document.

**OPUS: OP**timising patient o**U**tcomes by improving equity, cost effectiveness, and patient **S**election. OPUS refers to the Centre for Research Excellence in Total Joint Replacement, funded by the National Health and Medical Research Council and based at the Department of Surgery, University of Melbourne.

**Proxy:** Proxy describes a person who may be allowed to act for or represent someone else in certain circumstances. For example, a person sitting in a committee may be able to have a substitute person to fill in when they cannot attend the meeting themselves.

**Background**

This Terms of Reference document has been developed and informed by interviews with Aboriginal and/or Torres Strait Islander people who have expertise in Aboriginal health and health services (including Aboriginal patient liaison, general practice, nursing, chronic disease education and physiotherapy), Aboriginal health research as well as Aboriginal and Torres Strait Islander Elders and consumers who experience arthritis and total joint replacement. This document will be developed into a formal Terms of Reference in collaboration with the final ECRG.

**Our Story in a Painting**

Artist Mick Harding tells the story of our research through a scar tree and the diamonds within it, very much focusing on the relationship building and relationships within our musculoskeletal health research. The diamonds represent Liwik (ancestors). Aboriginal and Torres Strait Islander communities around Australia are represented by the designs alongside both sides and the limbs of the scar tree. The wavy lines at the top and bottom of the tree represent the overall journey of the research group and how its research has a ripple effect that influences possible change within our communities. The five colourful gum leaves represent the five difference research streams conducted at OPUS.

**
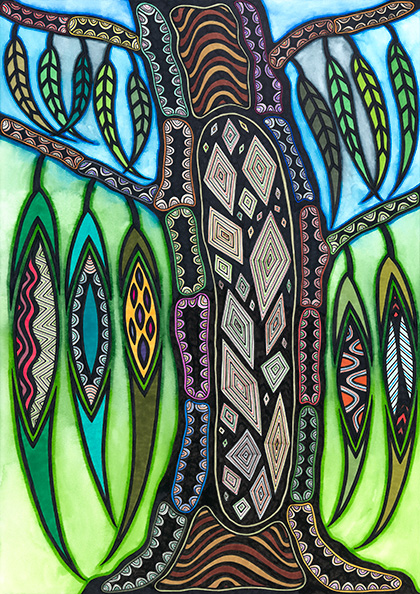
**

**Structure**

**
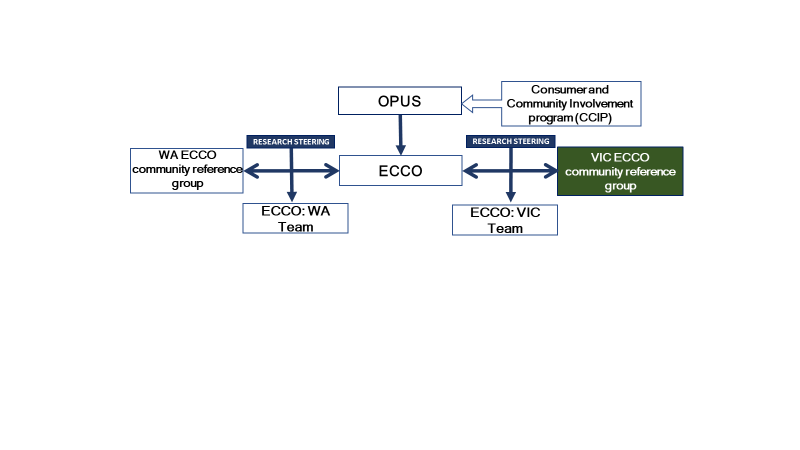
**

**Purpose**

The purpose of the ECRG is to provide the ECCO Collaborative guidance and advice in relation to all aspects of their Aboriginal health research activities. This includes ECCO’s function of promoting and facilitating research and knowledge sharing in relation to the musculoskeletal health and care of Aboriginal and/or Torres Strait Islander people. The ECRG will work in collaboration with the ECCO research team to navigate the research methods, processes, findings and dissemination of outcomes. The overall aim is for cultural security and cultural safety to be upheld in all ECCO research activities which should ultimately benefit Aboriginal and/Torres Strait Islander people who experience arthritis.

**Role**

The ECRG will discuss and decide upon their own role and responsibilities to the ECCO research project which will likely include facilitating community engagement, research steering, knowledge communication, dissemination and advocacy.

**Community engagement** aims of the ECRG are:

- To build meaningful engagement with the community which ECCO research aims to serve.
- To ensure that engagement with Aboriginal and/or Torres Strait Islander communities is not taken for granted, and that ECCO respects the input and cultural perspectives of ECRG members.
- To act as an individual voice from their respective communities, that provides advice to the research project on what, in their opinion, might best reflect the needs of Aboriginal and/or Torres Strait Islander communities.
- To give voice to the communities that the members represent, whilst also acknowledging and celebrating the diversity of culture within Aboriginal and/or Torres Strait Islander communities.

**Research steering** aims of the ECRG are:

- To act as navigators, drawing on their unique experiences and expertise to guide all cultural and methodological aspects of ECCO research.
- To assist with research priority setting for Aboriginal and/or Torres Strait Islander people who experience arthritis or joint pain.
- To provide advice and direction on how best to ensure that the work of ECCO is conducted in a culturally safe and secure manner.
- To provide advice and direction on how best to ensure that the work of ECCO is conducted in an ethical manner.
- To guide health care services and clinicians in providing musculoskeletal care and achieve positive health and wellbeing outcomes for Aboriginal and Torres Strait islander people who experience arthritis/joint pain.

**Knowledge communication and advocacy** aims of the ECRG are:

- To ensure that ECCO research findings are appropriately communicated to the communities they aim to benefit.
- To support and contribute to the development of mechanisms to raise awareness of ECCO work so that we can raise the profile of musculoskeletal health.
- To create a safe space for community members and support a two-way knowledge exchange between ECCO researchers and Aboriginal and/or Torres Strait Islander health services, patients and communities.

**Values**

The values of the ECRG will be decided upon collectively by the group. The ECRG may choose to work with ECCO’s working values of capacity building, trust, voice, honesty, benefit, flexibility, commitment, respect.


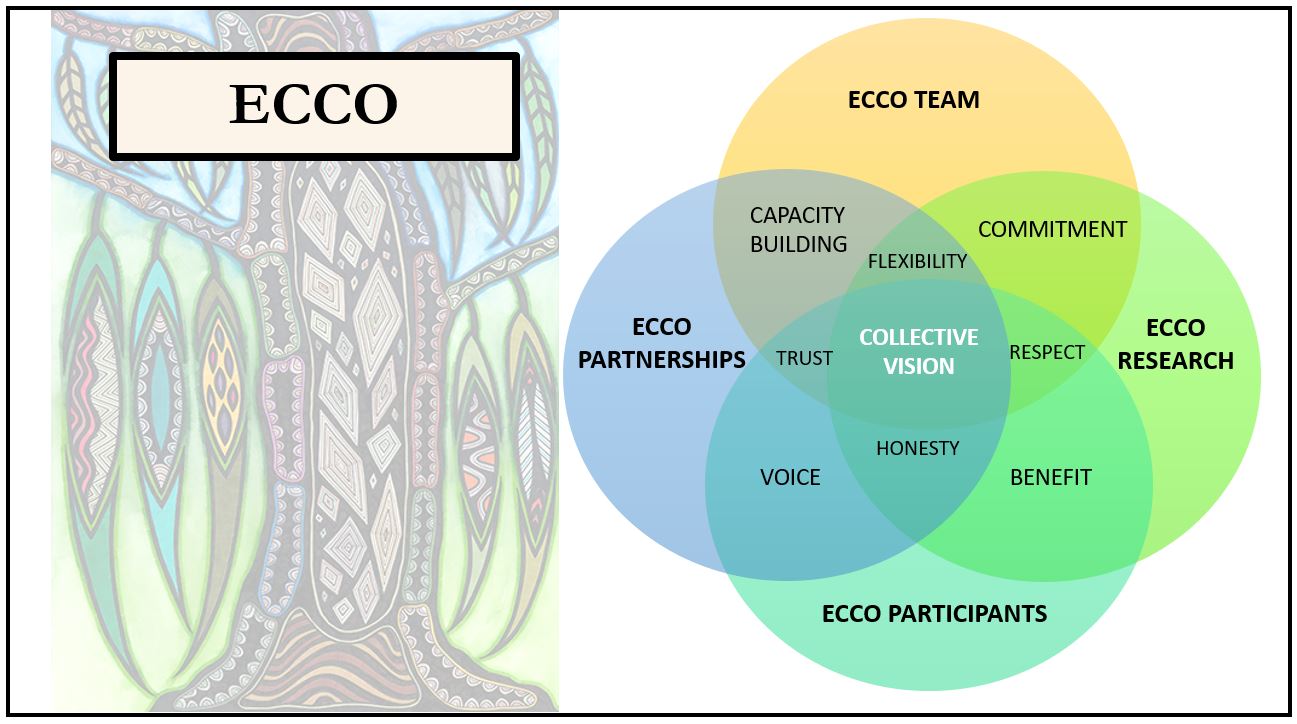


**Reporting**

The ECRG will report to the ECCO research group. Recommendations will be communicated by secretary/co-chair of the group to the ECCO research group at ECCO’s ongoing fortnightly meetings on Monday’s 11-11.30.

**Authority**

The ECRG is a reference group. The group will make recommendations to the ECCO research group, however does not provide final authority on decision making for policies. Final approval rests with the ECCO research team, on consultation with other project leaders. Although the ECRG does not have final decision-making authority, the ECRG will have a strong input in research steering. All recommendation outcomes will be communicated back to the ECRG with full transparency as to the outcomes reasoning.

**Membership**

***Composition***

The ECRG will consist of 4-10 Victorian members who will represent diversity in gender, age, geographical location, knowledge and expertise. The majority (80% or 8 out of 10 people) of the group’s members must identify as Aboriginal and/or Torres Strait Islander. Members of the group will have experience in and/or expertise in osteoarthritis and musculoskeletal care and prevention, health services, research and/or advocacy for Aboriginal and/or Torres Strait Islander people. Members will also have a strong connection to their community. The ECCO research group will provide secretarial support to the ECRG. A chair and co-chair will be appointed to the ECRG and will convene meetings. A quorum of four members will be required for a meeting. A quorum is the minimum number of members required at each ECRG meeting that must be present for the proceedings of the meeting to be valid.

***Members***

The ECRG will comprise of:

- Aboriginal Elder within the community.
- Aboriginal and/or Torres Strait Islander consumer with arthritis/joint pain (male).
- Aboriginal and/or Torres Strait Islander consumer with arthritis/joint pain (female).
- An Aboriginal and/or Torres Strait Islander family member of someone who has lived experience of arthritis/joint pain.
- A representative from a local Aboriginal and/or Torres Strait Islander health service.
- A representative from the St Vincent’s Hospital Melbourne Aboriginal Health Unit.
- A clinician with expertise in musculoskeletal health care and experience treating Aboriginal and/or Torres Strait Islander patients.
- An Aboriginal health researcher who has expertise in Indigenous research methodologies.
- An ECCO researcher.

Some members of the ECRG may be able to fulfill or represent more than one of the above-mentioned memberships.

***Proxy***

Proxy describes a person who may be allowed to act for or represent someone else in certain circumstances. For example, a person sitting in a committee may be able to have a substitute to fill in when they cannot attend. A member of the ECRG can nominate an Aboriginal and/or Torres Strait Islander person to act as a proxy when unable to attend a meeting. This proxy can be a work colleague or known individual who has experience in and/or expertise in osteoarthritis and musculoskeletal care and prevention, health services, research and advocacy for Aboriginal and/or Torres Strait Islander people. If members are to nominate a proxy, they must represent the same area of expertise within the group as the original member. If required, a proxy can vote if given authorization from the sitting member they are acting as proxy for. In this instance, the decisions made and their outcomes should be clearly communicated.

***Roles***

Members of the ECRG may be assigned specific roles within the group. Roles include Chair and Co-Chair, Secretary (take minutes, set up meetings and setting reminders) and Community liaison (communication to local Aboriginal health services and community groups in Victoria).

***Chairperson / Governance***

The Chair will be nominated and appointed by the ECRG and the position must be held by an Aboriginal and/or Torres Strait Islander identifying person who has had previous experience in committee membership. The role of the chairperson is to ensure that ECRG meetings are conducted in accordance with the Terms of Reference which will be agreed upon by all members of the group. The chair will also ensure that meetings are run in accordance with the collective values and expectations of the group which are outline in this Terms of Reference document. The Chair will hold the position for a minimum of one year. The Chair will also act as the primary contact for information flow between the ECCO research team and the members of the ECRG.

***Co-Chair***

The Co-chair will also be nominated and appointed by the ECRG. The Co-Chair will share the same responsibilities as the Chair. The Co-Chair may be a member of the ECCO research team to act as a liaison between the two groups. The ECRG will aim to appoint a male- and female-identifying Chair and Co-Chair.

***Responsibility***

All roles and responsibilities will be agreed upon collectively by the ECRG. Members of the ECRG are expected to:

- Work collaboratively and inclusively, acknowledging and respecting all members of the group who represent diverse communities and experiences and allowing all members to voice their opinion.
- Share their unique strategic and cultural advice, knowledge and experiences with the group to inform timely decisions and guide ECCO projects.
- Be committed to upholding the ECCO’s shared values of capacity building, trust, voice, honesty, benefit, flexibility, commitment, respect OR uphold the values decided upon by the ECRG.
- Be committed to upholding the principle of cultural security in all functions of the group.
- Demonstrate a commitment to privileging Aboriginal and/or Torres Strait Islander voices.
- Demonstrate a commitment to responding to Aboriginal and/or Torres Strait Islander identified priorities, to improve access to culturally secure musculoskeletal care.
- Attend meetings with an understanding of the forthcoming agenda and matters arising and therefore being able to proactively participate in meetings and respond in reasonable out of session requests.
- Fulfill individual roles and responsibilities in a timely manner.
- Where appropriate, take responsibility in communicating relevant news and finding to their communities, in the most accessible format possible (Facebook, newsletters, yarning circles, and posters).

***Expectations***

Members of the ECRG can expect:

- To be provided with complete and accurate information in a timely manner.
- To receive ongoing and up to date communication through a medium most accessible to all members (ECRG Facebook group, email and newsletters).
- To be given a reasonable timeframe to make decisions.
- To feel culturally safe and secure in all meetings and for appropriate channels to be available for members to voice concerns confidentially.

***Time Commitment***

The ECRG will be convened bi-monthly for the first 6 months upon the formation of the group or as mutually agreed. ECRG meetings will move to quarterly thereafter however will be informed by the intensity of the research projects and the capacity of the members of the group. There will also be the potential for shorter ad-hoc teleconferences as necessary. Ad-hoc meetings may not need to be attended by all members of the group. Members are appointed membership for a minimum of one year. As well as extending an invitation to individuals to participate in the ECRG, upon acceptance we will offer to write to the member’s employers and representative organisations to seek their support and acknowledge their contribution to the University of Melbourne through the release of the ECRG member for reference group activities and meetings. ECCO will also offer to write letters of recommendation or reference for members of the ECRG.

**Meeting process**

***Decision making***

The ECRG will adopt an informal consensus decision making processes to reach agreement at meetings. This will involve a collaborative, transparent approach in which all members have the opportunity to voice their opinions. Members of the group should feel safe and respected to voice their concerns in meetings. We recognise that at times members may not feel comfortable to raise concerns. In these circumstances, members will be able to confidentially contact the Chair or Co-Chair out of session. For sensitive topics, the ECRG may consider an anonymous decision-making process, for example using a paper ballot system. If a vote is required, consensus is reached with “half +1” of total present members.

***Meeting process and documents***

The meeting process and agenda follows a standard format in keeping with the agreed purposes of the ECRG. The standard format includes the following headings:

- Acknowledgement of country
- Check in / sorry business
- Minutes of the previous meeting
- Matters arising
- New projects
- ECCO feedback report
- Other business
- Next meeting
- Close

Members are encouraged to be mindful of potential sensitivities around any sorry business. Check in / sorry business is to be omitted from the minutes due to respect, privacy and sensitivity. The ECRG Secretariat will manage and provide documentation (e.g., agendas and minutes of meetings, relevant project updates, new project descriptions, documents for review) relevant to the ECRG. The agenda for each meeting and any required additional reading will be provided to ECRG members at least one week before each meeting via email and through the ECRG Facebook Group. The ECRG Secretariat will confirm best mode of contact with each individual group members and documentation can be arranged via alternatives which do not require access to the internet if required.

***Meeting Venue***

With the exception of the first meeting of the group which will be face-to-face, all meetings will be available to be joined via teleconferencing platform Zoom. Links to Zoom meetings will be circulated with all other meeting documentation via calendar invites, email and the ECRG Facebook group. Members will also be able to join in person at the Department of Surgery if possible and pending COVID restrictions. The meeting room is located at Level 2, Clinical Sciences Building, 29 Regent St Fitzroy, St Vincent’s Hospital Melbourne.

***Sitting fees***

All ECRG members will be remunerated for their time sitting at the meetings, and reimbursement for any out of pocket travel expenses in-line with the OPUS Policy for Reimbursement and Sitting Fees. A sitting fee of $100 per meeting will be offered to members as financial reimbursement. Light refreshments will also be offered if face to face meetings are to be arranged.

**Approval/Review Date**

This Terms of Reference document is a draft only and will be amended and formalized through consensus discussions at one or more of the initial ECRG meetings. The Terms of Reference will be reviewed at least annually, and changes or adjustments made accordingly in collaboration with the ECCO research team.

**References**

Ramsden, Irihapeti. (1992). Teaching cultural safety. *NZ Nursing Journal*, June, pp 21-23

Coffin, Juli. (2007). Rising to the Challenge in Aboriginal Health by Creating Cultural Security. *Aboriginal & Islander Health Worker Journal*, 31(3), pp 22-24
